# The Anti-Inflammatory Roles of Vitamin D for Improving Human Health

**DOI:** 10.3390/cimb46120807

**Published:** 2024-11-26

**Authors:** Aysen Kutan Fenercioglu

**Affiliations:** Department of Family Medicine, Cerrahpasa Medical Faculty, Istanbul University-Cerrahpasa, Istanbul 34098, Turkey; aysen.fenercioglu@iuc.edu.tr; Tel.: +90-5379645751

**Keywords:** vitamin D, inflammation, autoimmune disease, immune activation

## Abstract

Vitamin D receptors (VDRs) are present in almost all cells of the immune system, including B cells, T cells, NK (Natural Killer) cells, dendritic cells, and monocytes, as well as the epithelial cells of many organs such as the intestine, pancreas, prostate, lungs, and cardiomyocytes. In addition, some immune cells, including dendritic cells, macrophages, and B and T cells, can synthesize calcitriol by expressing 1α-hydroxylase. Upon binding to VDRs, vitamin D (Vit D) regulates the expression of genes involved in immune responses, including those encoding for cytokines. It modulates the production of pro-inflammatory cytokines while promoting the synthesis of anti-inflammatory cytokines. Vit D also affects the differentiation and maturation of cells of the immune system. By inhibiting the nuclear factor kappa B (NF-κB) and mitogen-activated protein kinase (MAPK) pathways, Vit D reduces the expression of pro-inflammatory genes. These effects highlight the potential of Vit D as a therapeutic agent in the management of inflammatory diseases, including autoimmune disorders, cardiovascular diseases, diabetes, metabolic syndrome, cancer, neurological diseases, depression, and inflammatory bowel disease.

## 1. Introduction

Vitamin D (Vit D) is a fat-soluble vitamin that plays an important role in calcium metabolism and bone health, but its role goes beyond the traditional role. After the discovery of the presence of the Vit D receptors (VDRs) and the enzymes that activate Vit D in many cells other than cells related to bone and mineral metabolism, the non-classical effects of Vit D have been proven by many scientific studies [1,2,3]. There are two types of Vit D (calciferol): vitamin D2 (ergocalciferol) and vitamin D3 (cholecalciferol) [4]. Vitamin D2 is obtained from foods (sun-grown mushrooms, fortified foods, and dairy products), while vitamin D3 is obtained from animal foods (fish, liver, margarine, milk, eggs, meat). Vitamins D2 and D3 are then absorbed by the small intestine via passive transport. The bioequivalence of the different forms of Vit D has been questioned in many studies and Vit D2 has been found to be either equivalent or slightly less potent than Vit D3 for obtaining optimal serum 25-hydroxyvitamin D concentrations, at least when given in daily dosages. However, when given intermittently, Vit D2 is clearly less potent than Vit D3 [5,6,7]. Vit D is also produced from the skin upon exposure to ultraviolet B (UVB) radiation. Vit D obtained from the skin and diet and is metabolized in the liver to 25-hydroxyvitamin D, which is used to determine a patient’s Vit D status. 25-Hydroxyvitamin D is metabolized in the kidneys by 25-hydroxyvitamin D-1α-hydroxylase (CYP27B1) to its active form, 1,25-dihydroxyvitamin D (calcitriol) [1,4].

The role of Vit D in human health has garnered significant attention in recent years, particularly regarding its anti-inflammatory properties [3,4,8,9,10,11,12]. Inflammation is defined as a host reaction to infections and tissue damage, but many studies have shown that it also plays a role in the pathophysiology of chronic diseases [4,8,9,11,12]. The relationship between Vit D and inflammation has been the subject of extensive investigation in recent years [8,9,10,11,12].

This literature review aims to synthesize existing research on the anti-inflammatory roles of vitamin D, elucidating its mechanisms of action, clinical implications, and potential therapeutic applications.

## 2. The Roles of Vit D in Diseases

VDRs and the Vit D activating enzyme 1-α-hydroxylase (CYP27B1) are expressed in many cell types such as intestine, pancreas, prostate, dendritic cells, and cells of the immune systems (T cells, B cells, macrophages) as well as bone and mineral tissue [1,4]. The active form of Vit D binds to VDRs, forming a ligand–receptor complex. This complex binds to specific DNA sequences called Vit D response elements (VDREs) located in the promoter regions of target genes [1,2,3]. Binding to VDREs initiates gene transcription, leading to the production of proteins that mediate the anti-inflammatory effects of Vit D. The Vit D-VDR complex modulates the immune response by influencing the differentiation and function of various immune cells, such as T cells, B cells, dendritic cells, and macrophages [1,4]. They also regulate cell growth and differentiation in various tissues, including skin, breast, and prostate, playing a role in carcinogenesis [3].

The role of Vit D against infectious agents is to enhance the differentiation of macrophages, which in turn release cytokines and phagocytose bacteria [3]. In addition, by binding to the “anti-microbial peptides”, Vit D enhances the “microbicidal activity” of phagocytes [12]. Vit D has been shown to induce the transcription of anti-microbial peptides—such as cathelicidin and defensin β2—in different human cell lines, including myeloid cells, monocytes/macrophages, and neutrophils [3,12].

Many studies have shown that Vit D can regulate the production of pro-inflammatory cytokines such as tumor necrosis factor-α (TNF-α), interleukin-6 (IL-6), interferon gamma, and interleukin-1β (IL-1β) while stimulating the synthesis of anti-inflammatory cytokines such as interleukin-10 (IL-10) [8,9,10,11,12,13]. Vit D inhibits the nuclear factor kappa B (NF-κB) and mitogen-activated protein kinase (MAPK) pathways and reduces the expression of pro-inflammatory genes [8,10]. These effects suggest the potential of Vit D as a therapeutic agent in the management of inflammatory diseases, including autoimmune disorders, cardiovascular diseases, diabetes, metabolic syndrome, cancer, neurological diseases, depression, inflammatory bowel disease, and respiratory conditions like chronic obstructive pulmonary disease (COPD) and asthma [8,11,13,14,15].

Vit D can also prevent chronic diseases by controlling oxidative stress in various ways such as inducing the expression of molecules involved in antioxidant protection, including glutathione, glutathione peroxidase, and superoxide dismutase (SOD), and inhibiting the expression of NADPH oxidase [13,15]. The antioxidant effects of Vit D also play important roles in chronic diseases. But this review article aimed to focus on the anti-inflammatory effects of Vit D rather than its antioxidant effects.

## 3. The Anti-Inflammatory Roles of Vit D in Autoimmune Diseases

Autoimmune diseases, characterized by dysregulated immune responses and chronic inflammation, have been particularly highlighted in the context of Vit D deficiency. Studies show that individuals with autoimmune diseases such as rheumatoid arthritis (RA), systemic lupus erythematosus (SLE), ankylosing spondylitis, psoriatic arthritis (PsA), Sjögren’s disease and inflammatory myopathies, inflammatory bowel disease (IBD), Crohn’s disease, ulcerative colitis, Hashimoto’s thyroiditis, type 1 diabetes (T1D), and neurodegenerative diseases such as Alzheimer’s (AD), Parkinson’s disease (PD), and multiple sclerosis (MS) often have lower Vit D levels [15,16,17,18,19,20,21,22,23,24,25,26,27,28,29,30,31]. Table 1 shows the anti-inflammatory effects of Vit D in autoimmune diseases.

In RA, Vit D levels were found to be associated with disease activity [17,18]. In SLE, it is shown that Vit D is inversely associated with disease activity and renal involvement [17,21,22]. Vit D deficiency may be associated with the development of neuropathy and lymphoma in Sjögren’s disease [19]. Most studies on Vit D supplementation have shown benefits in preventing or treating the effects of autoimmune diseases [23,24,29,31]. Vit D deficiency in IBD results from the inability to form and absorb Vit D micelles and chylomicrons in the intestine, increasing the risk of osteomalacia and osteoporosis [21]. Decreased Vit D levels are a biomarker of IBD activity and predictive of poor patient outcomes. In MS, Vit D supplementation shows a synergistic effect when combined with interferon β [22]. Barghava et al. showed that Vit D supplementation reduced oxidative stress (γ-glutamyl amino acids, glutathione) and caused alterations in xenobiotic metabolism (benzoate, caffeine) in patients with MS [23]. Most studies have shown that serum levels of IL-10 and transforming growth factor (TGF)-β1 increased after Vit D supplementation, and some have reported that disability scores were reduced after Vit D supplementation in MS patients [29].

All immune cells express VDR and are therefore sensitive to calcitriol-mediated regulation. Additionally, some immune cells, including dendritic cells, macrophages, and B and T cells, can produce calcitriol by expressing 1α-hydroxylase [1,4,21,22]. Vit D can affect the maturation and migration of different dendritic cell subtypes and their production of cytokines and chemokines [1,21]. The interaction of Vit D with VDR prevents dendritic cell differentiation and maturation [14,15,16,17]. This increases their tolerogenic state and reduces the production of pro-inflammatory cytokines (IL-6, IL-12, IL-23) and TNF-α [25,29]. Furthermore, it promotes the production of anti-inflammatory cytokines (IL-8, IL-10) and down-regulates the expression of major histocompatibility complex classes I and II and surface molecules (CD40, CD80, CD83, CD86) [2,21,22]. One of the key mechanisms through which Vit D impacts the immune response is promoting the differentiation of Tregs. Tregs are a specialized subset of immune cells that play a crucial role in maintaining immune tolerance [21]. Tregs act as suppressors of inflammation, preventing an overactive immune response. Vit D prevents B cells from differentiating into plasma cells and producing antibodies [21,22]. Vit D may also reduce T helper (Th) cell differentiation and proliferation [2,21,22]. In particular, Th1 and Th17 subpopulations are reduced, and Th2 differentiation is promoted in CD4+ T cells, producing IL-4. Tregs play a crucial role in suppressing the activation and function of Th1 and Th17 cells [2,13,19]. These effects on immune cells may explain the pivotal role of Vit D in the pathogenesis of various autoimmune diseases. Moreover, clinical trials have suggested that Vit D supplementation may reduce disease activity and improve clinical outcomes in patients with autoimmune diseases [16,21,22,23,24,29,30,31].

## 4. The Anti-Inflammatory Roles of Vitamin D in Cardiovascular Diseases

The cardiovascular system is another area where Vit D’s anti-inflammatory effects have been investigated. Table 2 shows the anti-inflammatory effects of Vit D in cardiovascular diseases. Chronic inflammation is a well-established contributor to the development of atherosclerosis and other cardiovascular diseases. Atherosclerosis, a chronic inflammatory disease characterized by plaque accumulation in arterial walls, is a leading cause of cardiovascular events such as heart attacks and strokes [13,32]. Traditionally, atherosclerosis has been known to result from the retention of lipoproteins, primarily low-density lipoprotein (LDL) cholesterol, in the intimal space of arteries. The retained LDL is modified and taken up by scavenger receptor-mediated phagocytosis, forming foam cells, which are cholesterol-laden cells that contribute to forming fatty plaques. This process results in the continued growth of inflammatory fatty infiltrates rich in leukocytes that appear macroscopically as plaques [32]. Vit D deficiency has been linked to markers of subclinical atherosclerosis, such as increased carotid intima-media thickness and coronary artery calcification [33]. These findings suggest that Vit D plays a significant role in the pathogenesis of atherosclerosis [13,33]. Endothelial dysfunction, characterized by impaired nitric oxide (NO) bioavailability, increased oxidative stress, and increased pro-inflammatory signaling, plays a crucial role in the early stages of atherosclerosis [13,33,34,35,36,37,38,39]. Vit D shows its anti-inflammatory effects by suppressing the production of pro-inflammatory cytokines such as TNF-α and IL-6 [8,9,10,11,12,13]. These cytokines are known to contribute significantly to the pathogenesis of atherosclerosis by promoting endothelial dysfunction, facilitating the recruitment of leukocytes to arterial walls, and facilitating the formation of foam cells within atherosclerotic plaques [32]. Additionally, VDR activation in endothelial cells results in the inhibition of NF-κB gene expression, leading to down-regulation of pro-inflammatory and pro-thrombogenic cytokines such as IL-6, as well as the up-regulation of thrombomodulin and IL-10 [34,35]. Vit D suppresses vascular calcifications and prevents the development of atherosclerotic plaques by inhibiting the transformation of macrophages into foam cells [35,37,39]. Vit D also suppresses the expression of adhesion molecules on endothelial cells, thereby reducing the adhesion and migration of leukocytes into the arterial walls [13,35].

Furthermore, Vit D reduces inflammation and stabilizes atherosclerotic plaques by promoting the differentiation of Tregs and suppressing the activity of Th1 and Th17 cells [36]. Additionally, Vit D supports the production of NO, an important molecule that regulates vascular tone and maintains optimal blood flow. NO acts as a vasodilator, helping to relax blood vessels and improve endothelial function. Vit D counteracts the negative effects of endothelial dysfunction by increasing NO production, thereby supporting the maintenance of healthy blood vessel function [13,35,37]. Oxidative stress is a major contributor to endothelial dysfunction, causing damage to the endothelial wall and impairing the ability to regulate blood flow and vascular health. The antioxidant effects of Vit D protect endothelial cells from oxidative damage, maintaining their functionality and promoting vascular health [13,33,34,35].

Dyslipidemia, characterized by imbalances in cholesterol levels, particularly elevated LDL cholesterol and reduced high-density lipoprotein (HDL) cholesterol, is a major risk factor for the development of atherosclerosis. Vit D has been shown to have beneficial effects on lipid metabolism. It promotes the expression of specific genes involved in cholesterol efflux, such as ATP-binding cassette transporter A1 (ABCA1). ABCA1 is crucial in removing cholesterol from foam cells, which are key players in forming atherosclerotic plaques [13,38]. Thus, Vit D promotes cholesterol efflux from the endothelium, stabilizing the plaques. Stable plaques are less prone to rupture and thrombosis, reducing the risk of cardiovascular events such as heart attacks and strokes. Furthermore, Vit D can influence the expression and activity of enzymes involved in lipid metabolism, such as hepatic lipase and lipoprotein lipase, helping to maintain a favorable lipid profile [13]. Through the limitation of scavenger receptor expression on the macrophage surface, Vit D reduces LDL uptake by macrophages [39].

Hypertension, a major risk factor for cardiovascular events, is influenced by multiple factors, including vascular tone, renal function, and the renin–angiotensin–aldosterone system. Vit D plays a role in blood pressure regulation through several mechanisms. It inhibits the renin–angiotensin system, promotes the production of vasodilatory molecules such as NO, and regulates calcium homeostasis in vascular smooth muscle cells [35,37,39]. Vit D appears to decrease the activity of the cyclic adenosine monophosphate response element in the renin gene promoter [13]. Vit D deficiency has been associated with an increased risk of hypertension, whereas Vit D supplementation has shown potential benefits for lowering blood pressure. Vit D promotes proper vasodilation and regulates blood flow by improving endothelial function, reducing oxidative stress, and modulating inflammatory pathways [13,33,34,35,36,37,38].

VDR and the enzyme 1-α-hydroxylase, necessary to form the active form of Vit D, are also found in ventricular cardiomyocytes. Upon binding to VDRs of cardiomyocytes, Vit D is activated and prevents the remodeling of cardiomyocytes by acting on genes that lead to heart failure. This mechanism utilized by Vit D is not always absolutely understood, but the evidence that VDR is expressed on cardiomyocytes suggests the activation of intracellular pathways, causing modified expression of hypertrophy-associated genes, such as myosin heavy-chain isoform, α-tropomyosin, and via the down-regulation of NF-κB [35,39,40,41]. Furthermore, Vit D activation stimulates calcium uptake, thus increasing contractility and improving diastolic contractility [35,39]. Vit D can also decrease extracellular matrix and fibrosis in cardiomyocytes by way of interfering with collagen and metalloproteinase synthesis [35,39,40,41].

Atherosclerosis, closely linked with inflammation, affects both the initiation and progression of coronary artery disease. Vit D can prevent coronary artery disease by its positive effects on atherosclerosis, hypertension, and dyslipidemia. These effects are mainly achieved by reducing the expression of IL-1, IL-6, and TNF-α, limiting the formation of macrophage-derived foam cells, and reducing LDL uptake by macrophages, as well as by left ventricular remodeling [39,40,41]. There is an association between low Vit D levels and an increased risk of acute myocardial infarction, coronary heart disease, angina pectoris, and stroke [39,40].

Strong epidemiological proof links Vit D deficiency to the elevated risk of cerebrovascular diseases, including ischemic stroke [41,42,43]. Vit D deficiency is common in individuals who have suffered a stroke, which is generally associated with superior age, restrained mobility, reduced daylight exposure, and malnutrition. About 85% of strokes result from a clot formed in a cerebral artery in which a dysfunctional endothelium would be typically present [42,43]. Low Vit D levels contribute to about 50% increased risk of incident stroke [43]. There is also evidence that high serum levels of Vit D are associated with much less cognitive impairment among stroke patients [42]. Furthermore, there is evidence that sufficient levels of dietary Vit D ought to exert numerous neuroprotective actions, consisting of a reduction in oxidative stress, a decrease in neuronal inflammation and, eventually, dying from stroke [41,43]. The direct effects of Vit D deficiency in increasing the risk of stroke are increased platelet aggregation, increased tissue factor expression, decreased antithrombin and thrombomodulin expression, impaired synthesis of neurotrophic factors and neurotransmitters, and blocked detoxification pathways of the brain. In addition, Vit D deficiency is linked to hypertension and atherosclerosis, which are the major modifiable risk factors for cerebral ischemia [41,42,43].

Studies have shown that cardiovascular diseases, heart failure, and hypertension are associated with Vit D deficiency [39,40,41,42,43]. Despite overwhelming evidence to date, most clinical trials have failed to demonstrate benefits of Vit D supplementation in these diseases [3]. This inconsistency highlights the need for greater knowledge and understanding of vitamin D metabolism and cardiovascular effects to determine which patients may benefit from vitamin D supplementation [39].

## 5. The Anti-Inflammatory Roles of Vitamin D in Metabolic Diseases

Metabolic syndrome, characterized by a cluster of conditions including obesity, insulin resistance, and hypertension, has also been associated with low Vit D levels. Inflammatory processes play a central role in the pathophysiology of metabolic syndrome, and Vit D’s ability to modulate these processes has been explored in several studies [8,9,11,44,45,46,47,48,49]. Table 3 shows the anti-inflammatory effects of Vit D in metabolic diseases. Research has indicated that Vit D can improve insulin sensitivity and reduce inflammation in adipose tissue, suggesting a potential role in the prevention and management of metabolic disorders [9,44,45,47,48,49]. Defects in pancreatic β-cell function, decreased insulin sensitivity, and systemic inflammation are frequently present at the onset of glucose intolerance and type 2 diabetes mellitus (T2DM) [44,45,46]. Vit D exerts its effects by binding of its circulating active form to the β-cell VDR or by the formation of its active form by 1-α-hydroxylase enzyme within the β-cell [46]. The binding of Vit D with pancreatic VDR activates the genomic pathway in the insulin gene promoter region, leading to the stimulation of insulin synthesis [8]. The indirect effects of Vit D may be its role in the regulation of extracellular calcium and calcium flux through the pancreatic β-cells. Insulin secretion is a calcium-dependent process, and a lack of Vit D or insufficient calcium intake can change the balance between the calcium pools in the extracellular and intracellular cells. This can disrupt the normal release of insulin, especially in response to a high glucose load [46].

Vit D deficiency results in reduced levels of calcitriol to bind to VDR located on pancreatic β-cells, which impairs the insulin signaling pathway. Peroxisome proliferator-activated receptor delta (PPAR-δ) is deactivated as a result of this. PPAR-δ is a transcription factor that plays a pivotal role in the mobilization and breakdown of free fatty acids (FFAs) in both muscle and adipose tissue and prevents insulin resistance induced by FFAs [11,46]. As a result of the deactivation of PPAR-δ, the expression of insulin receptors (IRs) on the surface of these cells is decreased. Consequently, the liver, skeletal muscles, and adipose tissue are less able to absorb glucose. This leads to chronic hyperglycemia, which increases the production of reactive oxygen species (ROS) and causes oxidative stress [8,47]. Abnormal glucose metabolism and the development of insulin resistance are also linked to subclinical inflammation [44,45,48,49]. Vit D’s anti-inflammatory effects in metabolic diseases are again achieved by reducing the pro-inflammatory cytokines, such as TNF-α, IL-6, interferon-gamma, and IL-1β, and increasing the anti-inflammatory cytokines like IL-10 and IL-8 [8,9,10,46]. Likewise, Vit D has been shown to inhibit the NF-κB and MAPK pathways. thereby reducing the expression of pro-inflammatory genes in metabolic diseases [8,10,46,47].

Adipose cells are triggered to release a variety of pro-inflammatory cytokines that directly impede insulin signaling by certain stimuli, such as overeating and obesity. These adipo-cytokines show their effects by NFκB and the c-Jun NH2-terminal kinase (JNK)/AP-1 signaling pathways. Insulin signaling is disrupted and the expression of genes that are responsible for multiple inflammatory cytokines is impacted by the corruption of these signaling pathways [11]. Ding et al. investigated the effects of Vit D on signaling pathways and macrophage-mediated inflammation in cultured human adipocytes. The expression of the NFκB inhibitor kB-α (IkB-α) protein was significantly inhibited in MC (macrophage-conditioned) medium, which contained 25% adipocyte medium, and the levels of NFκB were elevated. IkB-α expression went up and NFκB phosphorylation went down when calcitriol was added to the medium. As a result, NFκB signaling activation induced by macrophages was inhibited [10].

T2DM and its complications are both caused and exacerbated by oxidative stress. High glucose concentrations activate reactive oxygen species (ROS) and NFκB pathways, and the inflammatory cytokines monocyte chemotactic protein-1 (MCP-1) and leukocyte interleukin-8 (IL-8) are secreted in response to this. Diabetes progression is sped up by elevated MCP-1 and IL-8 levels in the blood [10,46]. Serum ferritin levels are typically significantly elevated in T2DM patients, and individuals with elevated ferritin levels are more likely to develop T2DM. Excessive iron intake, particularly heme iron from red meat, causes oxidative damage to β-cells and impairs the suppression of hepatic glucose production. These effects of iron increase the risk of T2DM [8]. Divalent metal transporter 1 (DMT1) was thought to facilitate iron uptake in pancreatic cells, and NFκB activity controls DMT1 expression. By reducing the NFκB-DMT1 pathway, Vit D prevents iron accumulation in β-cells and improves islet morphology and β-cell function. By down-regulating phosphorylated p38 MAPK and phosphorylated ERK1/2, which are the conventional MAPKs, Vit D can also reduce MAPK signaling. A positive regulatory role for the ERK1/2 signaling module in glucose-stimulated insulin secretion in pancreatic β-cells was suggested by a number of studies [8,10,46,47].

## 6. The Anti-Inflammatory Roles of Vitamin D in Miscellaneous Diseases

Vit D deficiency is linked to a wide range of neuropsychiatric disorders and neurodegenerative diseases, according to epidemiological evidence [15]. Vit D deficiencies in adulthood have been linked to a variety of negative brain outcomes, including Parkinson’s disease (PD), Alzheimer’s disease, depression, and cognitive decline, whereas Vit D deficiencies early in life have been linked to neuropsychiatric disorders like schizophrenia. According to a number of animal and clinical studies [15,23,27,28,29,31], Vit D supplementation may have some “neuroprotective” effects for epilepsy, multiple sclerosis, PD, and chronic stress. A significant factor in the development of schizophrenia is Vit D deficiency during pregnancy. The VDR is found in areas of concern in schizophrenia (such as dopaminergic regions) and influences brain growth during fetal development [15,50]. Dopamine metabolism changes in Vit D deficiency, which may be a reflection of the characteristic decline in dopamine neuron expression found in schizophrenia [15].

Depression develops as a consequence of an alteration in neural activity, which involves the elevation in “glutamate”, which is an “excitatory neurotransmitter”, and the inhibition of “Gamma-Aminobutyric Acid (GABA)”, which is an “inhibitory neurotransmitter”. When GABA binds to GABA receptors, it has a calming and soothing effect [51,52]. On the other hand, its inhibition can cause stress, anxiety, and depression. This is due to the increased intracellular calcium levels in the inhibitory neurons, which cause neuronal apoptosis and necrosis. Vit D regulates calcium homeostasis in neurons. By maintaining calcium homeostasis and stimulating tryptophan, Vit D regulates serotonin levels, which are involved in controlling our mood and prevent depression [4,15].

Maternal/neonatal Vit D deficiency has been proposed as a possible environmental risk factor for autism spectrum disorders due to its involvement in early neurodevelopment, the immune system, and gene regulation processes. Individuals with autism have immune function defects such as elevated levels of inflammatory cytokines and ongoing inflammation in their brains, similar to those affected by Vit D deficiency [4]. Vit D activates the transcription of the serotonin-synthesizing gene tryptophan hydroxylase 2 in the brain and represses the transcription of tryptophan hydroxylase 1 in tissues outside the blood–brain barrier. This mechanism explains the low concentrations of serotonin in the brain and its elevated concentrations in tissues outside the blood–brain barrier in autism [53].

Vit D’s inhibition of malignant cell proliferation and enhancement of normal cell differentiation contribute to cancer prevention. Vit D also functions as an “antioxidant”, plays a great role in “immunity”, and lowers the risk of cancer [1,3,4,14]. But this highly depends on the blood calcitriol levels. According to one study, a 92.5 nmol/L serum concentration of calcitriol reduces the likelihood of colorectal cancer by 50% compared to those with a <15 nmol/L concentration of calcitriol in the blood [54]. An inverse relation between serum Vit D levels and stomach cancer has also been demonstrated [55]. One study also indicates that serum concentrations of calcitriol of about 52 ng/mL reduce the risk of breast cancer development by 50% [56].

The idea that Vit D and gastrointestinal malabsorptive conditions like celiac disease have a bilateral relationship is strengthened by the discovery that VDRs are highly expressed in intestinal epithelial cells and all cell populations of the intestinal wall. Vit D is now considered a factor that influences the pathogenesis of celiac disease in all of its various factors, as well as the disease’s outcome [57]. The Vit D activating enzyme 1-α-hydroxylase (CYP27B1) is expressed by cells in the intestine, pancreas, prostate, and immune system, in addition to those involved in bone and mineral metabolism [1,14,15,16,17,58]. Many of these epithelia also exhibit elevated CYP27b1 expression in the early stages of cancer. This suggests that Vit D has a greater impact on human health than previously thought [58]. The extra-renal synthesis of calcitriol by immune cells and peripheral tissues has been proposed to have immunomodulatory properties similar to locally active cytokines [58]. Vit D deficiency appears to play a significant role in both acute and chronic pancreatitis, according to recent research. These studies demonstrated that Vit D deficiency is associated with pancreatitis by its anti-inflammatory and anti-fibrotic effects upon binding to VDRs [59].

## 7. Conclusions

In conclusion, the anti-inflammatory roles of Vit D represent a promising area of research with significant implications for human health. The evidence supporting the association between Vit D status and various inflammatory conditions is compelling, suggesting that maintaining adequate Vit D levels may be crucial for preventing and managing diseases characterized by chronic inflammation [8,9,10,11,12,13,14,15]. The anti-inflammatory effects of Vit D play a role in many diseases including autoimmune diseases, neurodegenerative diseases, cardiovascular diseases, metabolic diseases, and cancer. Although Vit D functions with different anti-inflammatory mechanisms in each of these diseases, there are some mechanisms of action that are common to all.

Future research should focus on elucidating the precise mechanisms by which Vit D exerts its anti-inflammatory effects, as well as conducting well-designed clinical trials to establish optimal dosing strategies and treatment protocols. Moreover, public health initiatives aimed at increasing Vit D awareness and supplementation could play a vital role in improving overall health outcomes and reducing the burden of inflammatory diseases in the population.

## Figures and Tables

**Table 1:** The anti-inflammatory effects of Vit D in autoimmune diseases.

The Autoimmune Disease	The Effects of Vit D
Sjögren’s syndrome	Vit D deficiency was associated with leukopenia and the development of lymphoma in the context of Sjögren’s syndrome [16,20]. Vit D supplementation has a prophylactic role in these patients [16,22,30].
Autoimmune thyroid diseases	Vit D significantly reduces the levels of anti-thyroperoxidase antibodies [22,24]. There is a negative relationship of pro-inflammatory cytokine levels (IL-8, IL-10, IL-1 β, and TNF-α) with total Vit D levels [24].
Multiple sclerosis (MS)	Vit D deficiency in MS increases inflammation of the central nervous system through T helper cells, cytotoxic T lymphocytes (CTLs), B cells, and NK cells, damaging neurons and oligodendrocytes. There is a predisposition to develop MS in HLA-DRB1-positive individuals. Vit D response elements are found in the promoter region of the HLA-DRB1 gene [21]. Vit D supplementation was found to exert a synergic beneficial effect in combination with interferon β [22,27,29]. Metabolic alterations in oxidative stress (γ-glutamyl amino acid, glutathione) and xenobiotic metabolism (benzoate, caffeine) were observed after Vit D supplementation [23].
Alzheimer’s disease	Vit D inhibits the transcription of amyloid precursor protein (APP) and reduces the level of Aβ and hyperphosphorylated Tau protein by down-regulating the levels of MAPK-38p and ERK1/2. In addition, Vit D can act on calcium channels and improve the expression of calcium buffer and maintain calcium homeostasis, eliminate Aβ/Ca2+ positive feedback, increase neuronal excitability, and promote cell proliferation and neurogenesis [15,26]. Vit D is an antioxidant that regulates the detoxification processes of the brain by regulating the activity of γ-glutamyl transpeptidase. It also regulates the synthesis of neurotrophic factors that are important for neuronal survival [15].
Parkinson’s disease	Vit D plays a role in the regulation of tyrosine hydroxylase gene expression and dopamine biosynthesis [15]. Vit D treatment reduces dopaminergic neurodegeneration and neuroinflammation mediated through down-regulating the expression of iNOS and toll-like receptor 4 (TLR-4) and up-regulating the expression of anti-inflammatory cytokines (IL-10, IL-4, and TGF-β), as well as CD163, CD206, and CD204 [25,28]. Its effect of restoring calcium homeostasis is also beneficial [15,25,28,31].
Systemic lupus erythematosus (SLE)	Vit D significantly changes the serum levels of antibodies and pro-inflammatory cytokines [16]. A negative correlation was observed between Vit D and the inflammatory cytokines IL-17 and IL-23, as well as antinuclear antibodies. Low C3 and C4 levels may be related to low vitamin D levels [17,22]. Vit D has a protective role in lupus nephritis induced by autoantibodies [21].
Rheumatoid arthritis (RA)	Vit D deficiency is more frequent among patients with RA and may be a cause of its onset [18,19]. An inverse relationship was observed between Vit D and CRP [16,22]. Treatment with Vit D is considered beneficial due to Th1 and Th17 (T helper lymphocyte subpopulations) suppression.
Ankylosing Spondylitis	A reverse association exists between Vit D levels and disease activity. This may be related to the effects of Vit D in bone metabolism and inflammatory activity [16].
Systemic Sclerosis (SSc)	Low Vit D levels have been observed in patients with systemic sclerosis due to the presence of antivitamin D antibodies in 87% of SSc patients [21]. Vit D affects the fibrinogenic activity of fibroblasts by suppressing tissue growth factor (TGF) and it reduces fibrosis in these patients [22].
Inflammatory Myopathies	Low serum levels of Vit D have been observed in patients with idiopathic inflammatory myopathies, in particular polymyositis, dermatomyositis, inclusion body myositis, and juvenile dermatomyositis [22].
Crohn’s disease and ulcerative colitis	Vit D inhibits the production of Th1 and Th17 and inflammatory cytokines in the gastrointestinal tract [13,19,22]. It induces cathelicidin and IL-10, represses TNF-α, and suppresses Escherichia coli growth [16,21,22,23]. It also intervenes on intestinal mucosal cells by stimulating proteins involved in membrane junction integrity and intracellular pathogen recognition [21].
Type 1 Diabetes (T1D)	The mechanisms involved in T1D include the production of autoantibodies and autoreactive Th1 and CTL, resulting in immune-mediated destruction of insulin-producing pancreatic cells [22]. Vit D may stimulate insulin secretion in pancreatic β cells by directly binding to VDR and inhibiting Th1 activity and may play a protective role in the development of T1D [21]. Vit D supplementation promotes a decrease in daily insulin levels and an increase in C-peptide levels.
Psoriasis	Topical treatment with the Vit D analog calcipotriol modulates the expression of pro-inflammatory cytokines and proteins, thereby enhancing the inflammatory reactions [16,22]. However, further clinical studies are needed to demonstrate the efficacy of oral calcitriol in the treatment of psoriasis [21].

**Table 2:** The anti-inflammatory effects of Vit D in cardiovascular diseases.

The Cardiovascular Diseases	The Effects of Vit D
Atherosclerosis	Vit D inhibits the production of pro-inflammatory cytokines (TNF-α and IL-6), promotes the differentiation of Tregs, inhibits the activation and function of Th1 and Th17 cells, up-regulates the production of nitric oxide (NO), suppresses the expression of adhesion molecules on the endothelial wall, down-regulates the formation of foam cells, and protects endothelial cells from oxidative damage [13,33,34,35,36,37,38].
Lipid metabolism and plaque stability	Vit D promotes the expression of genes involved in cholesterol efflux, such as ATP-binding cassette transporter A1 (ABCA1); stabilizes plaques by inhibiting the formation of foam cells [38]; affects the expression and activity of enzymes involved in lipid metabolism, such as hepatic lipase and lipoprotein lipase [13]; and reduces LDL uptake by macrophages [39].
Blood pressure regulation	Vit D inhibits the renin–angiotensin system, promotes the production of vasodilator molecules such as NO, and maintains calcium homeostasis in vascular smooth muscle cells [35,37,39]. Vit D appears to reduce the activity of the cyclic adenosine monophosphate response elements in the renin gene promoter [13].
Heart failure	Vit D prevents cardiomyocyte remodeling by altering the expression of hypertrophy-related genes, such as myosin heavy-chain isoform, α-tropomyosin, and by inhibiting NF-κB [35,39,40,41]. Activation of the Vit D pathway increases calcium uptake, enhances contractility, and improves diastolic function [35,39].
Coronary artery diseases	Vit D reduces the expression of IL-1, IL-6, and TNF-α, limits the formation of macrophage-derived foam cells, reduces low-density lipoprotein cholesterol (LDL) uptake by macrophages, and encourages left ventricular remodeling [39,40].
Cerebrovascular diseases	Vit D deficiency increases platelet aggregation, up-regulates the expression of tissue factor, down-regulates the expression of antithrombin and thrombomodulin, impairs the biosynthesis of neurotrophic factors and neurotransmitters, and blocks detoxification pathways in the brain [41,42,43].

**Table 3:** The anti-inflammatory effects of Vit D in metabolic diseases.

The Metabolic Diseases	The Effects of Vit D
Insulin Resistance and prediabetes	Vit D regulates insulin signaling pathway and the expression of insulin receptors (IRs) on the surface of insulin-responsive cells. Vit D activates peroxisome proliferator-activated receptor delta (PPAR-δ), a transcription factor that plays an important role in the mobilization and breakdown of fatty acids in both muscle and adipose tissue, and may reduce insulin resistance induced by free fatty acids (FFAs), ultimately averting cellular insulin resistance [11,46]. Vit D has been shown to suppress the NF-κB and MAPK pathways, thereby reducing the expression of pro-inflammatory genes [8,10,46,47].
Obesity	Vit D increases insulin sensitivity and reduces inflammation in adipose tissue [9,44,45,48,49]. Vit D modulates inflammatory pathways such as NFκB and the c-Jun NH2-terminal kinase (JNK)/AP-1 signaling pathway and increases NF-κB inhibitor kB-α (IkB-α) protein expression [8,10].
Type 2 diabetes mellitus	Vit D has been shown to have a positive effect on insulin secretion by directly affecting VDRs and vitamin D-dependent calcium-binding proteins in β-cells, and indirectly by regulating calcium transport in β-cells [38]. Vit D inhibits macrophage-induced activation of NF-κB signaling, improves islet morphology and β-cell function by reducing iron accumulation via suppression of the NFκB-DMT1 pathway, and can attenuate the MAPK signal by down-regulating phosphorylated p38 MAPK and phosphorylated ERK1/2 [10,41]. Vit D regulates the production of pro-inflammatory cytokines, such as TNF-α, IL-6, interferon-gamma, and IL-1β while promoting the synthesis of anti-inflammatory cytokines such as IL-10 [8,9,10,11,12,13].
